# Exosomes multifunctional roles in HIV-1: insight into the immune regulation, vaccine development and current progress in delivery system

**DOI:** 10.3389/fimmu.2023.1249133

**Published:** 2023-10-27

**Authors:** Arslan Habib, Yulai Liang, Naishuo Zhu

**Affiliations:** ^1^ Laboratory of Molecular Immunology, State Key Laboratory of Genetic Engineering, School of Life Sciences, Fudan University, Shanghai, China; ^2^ Institute of Biomedical Sciences, School of Life Sciences, Fudan University, Shanghai, China

**Keywords:** drug delivery, exosomes, HIV-1, immune system, vaccine

## Abstract

Human Immunodeficiency Virus (HIV-1) is known to establish a persistent latent infection. The use of combination antiretroviral therapy (cART) can effectively reduce the viral load, but the treatment can be costly and may lead to the development of drug resistance and life-shortening side effects. It is important to develop an ideal and safer *in vivo* target therapy that will effectively block viral replication and expression in the body. Exosomes have recently emerged as a promising drug delivery vehicle due to their low immunogenicity, nanoscale size (30-150nm), high biocompatibility, and stability in the targeted area. Exosomes, which are genetically produced by different types of cells such as dendritic cells, neurons, T and B cells, epithelial cells, tumor cells, and mast cells, are designed for efficient delivery to targeted cells. In this article, we review and highlight recent developments in the strategy and application of exosome-based HIV-1 vaccines. We also discuss the use of exosome-based antigen delivery systems in vaccine development. HIV-1 antigen can be loaded into exosomes, and this modified cargo can be delivered to target cells or tissues through different loading approaches. This review also discusses the immunological prospects of exosomes and their role as biomarkers in disease progression. However, there are significant administrative and technological obstacles that need to be overcome to fully harness the potential of exosome drug delivery systems.

## 1 Introduction

Human immunodeficiency virus (HIV) is associated with acquired immunodeficiency syndrome (AIDS), which can be suppressed by antiretroviral drugs (ARVs) that hinder crucial stages in the HIV life cycle (1). However, these drugs could not target or remove the genetic material of viruses fused with the cellular genome. If viral genome incorporation occurs into cellular genetic material, it becomes very difficult to cure individuals because a small portion of HIV-persistent cells stays asleep or curbed at certain anatomic places in the whole body. HIV-infected cells can be treated with antiretroviral drugs within these reservoirs, which later become persistent or latent. HIV-infected cells have the potential to produce new virions, but the dormant cells cannot produce virions although they could synthesize viral transcripts and proteins or stay transcriptionally intact (2). However, upon reactivation or exposure to antiretroviral drug interference, they may start rebounding with viral loads of HIV to a significant percentage within 14 days (3). Resting memory CD4+ T-cells are the most well-known latently infected cells, and they can be classified into various subtypes (4). In CD4+ T-cells, HIV latency transforms their domain from an effector to an elongated resting memory state (5). HIV can incorporate its DNA into host genetic material and it might be possible that it infects during the transformation period. The mechanism for incorporation is random and may occur at multiple sites in one cell. The phenomenon of incorporation usually occurs when the host transcription factors (NF-κB, Sp1 and AP-1) seem unavailable. This integration is followed by the latency of cells in prolonged CD4+ T-cell memories. The half-life of CD4+ T-cell memories is usually shown to be about 44 months (6). Additionally, cells such as monocytes and macrophages can also provide shelter for viruses.

**Figure 1:**
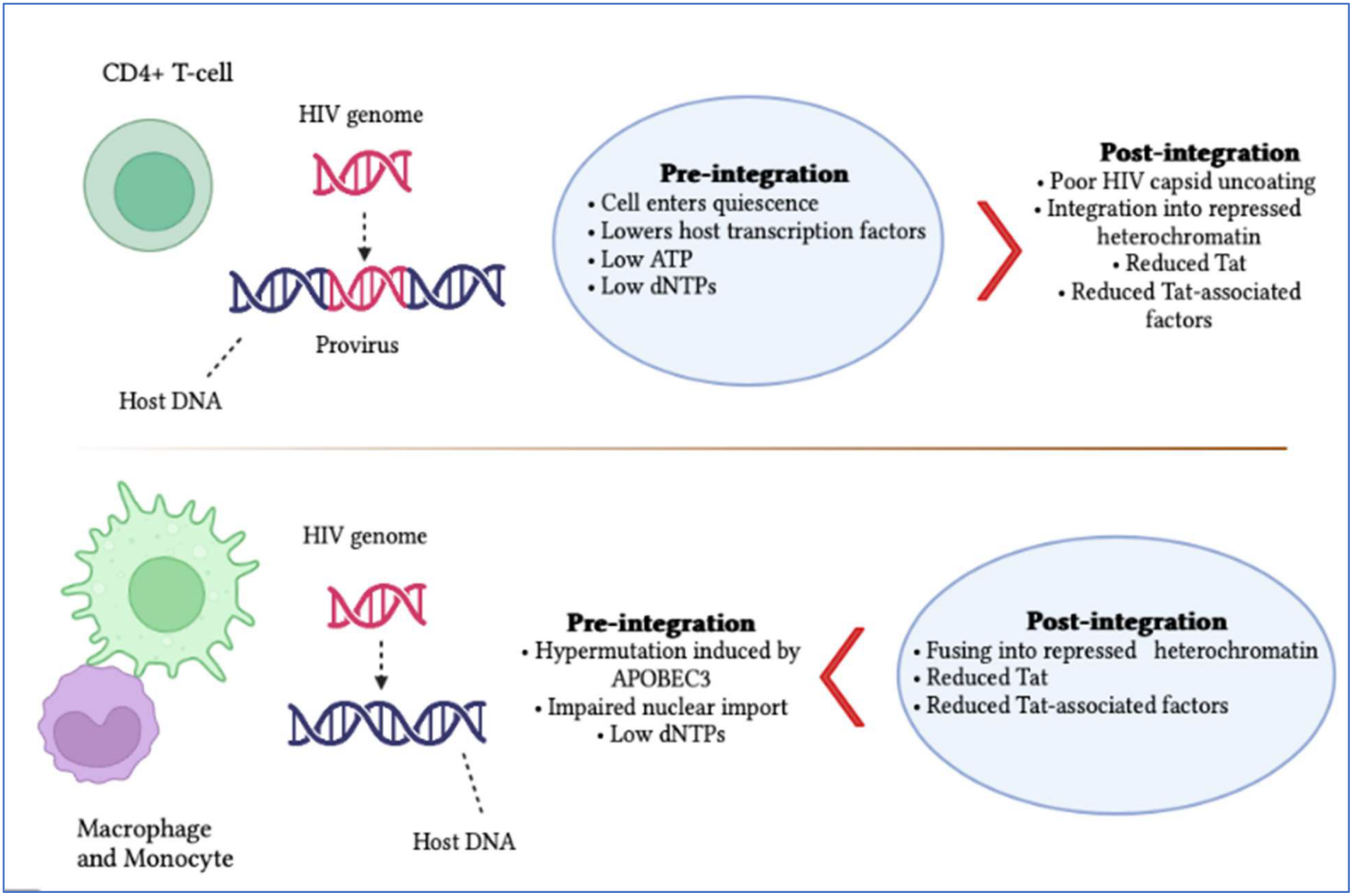
Summary of HIV pre- and post-integration latency. Imperfect nuclear transport of the pre-integration complex, inoperative reverse transcriptase and fluctuations in the cell cycle stage contribute to pre-integration latency, which is more commonly observed in monocytes/macrophages. Meanwhile, moderate cellular transcription factors, transcriptional intrusion, decreased p-TEFb and Tat protein and DNA methylation, as well as chromatin organization, contribute to post-integration latency in memory T-cells during antiretroviral treatment, which is only partial. The blue boxes indicate which category of latency is more frequent. It is also significant to note that pre-integration latency is more common in the T-cells of untreated individuals.

Latently persistent HIV-infected cells tend to gather at specific anatomical locations, such as the brain (7). These anatomical sites are shown to have two kinds of latency known as pre- and post-integration (**Figure 1**). The first type of latency, known as pre-integration latency occurs when the host cell enters a silent condition before the HIV DNA is incorporated. The pathway followed by this kind of latency involves the defective nuclear passage of the faulty viral protein as well as the stage of the life cycle and pre-integration latency (PIC) (8). Additionally, the PIC of the virus can stay on centrosomes for several weeks and it might be incorporated into the genetic material of the host when the reactivation of host cells occurs (9). Post-integration latency is more balanced than pre-integration. The phenomenon of post-integration latency arises after the integration of viral DNA material into the cellular genome followed by their transcriptionally silent stay (10). The transformation of active memory CD4+ T-cells to a dormant phase also exhibits post-integration latency. Many factors can be responsible for this phase including transcriptional suppression, DNA modification, low Tat and P-TEFb and low transcriptional factors. Identifying and amplifying the anatomical reservoirs of HIV is a challenging task for current research as many of these sites are distributed throughout the body.

**Figure 2:**
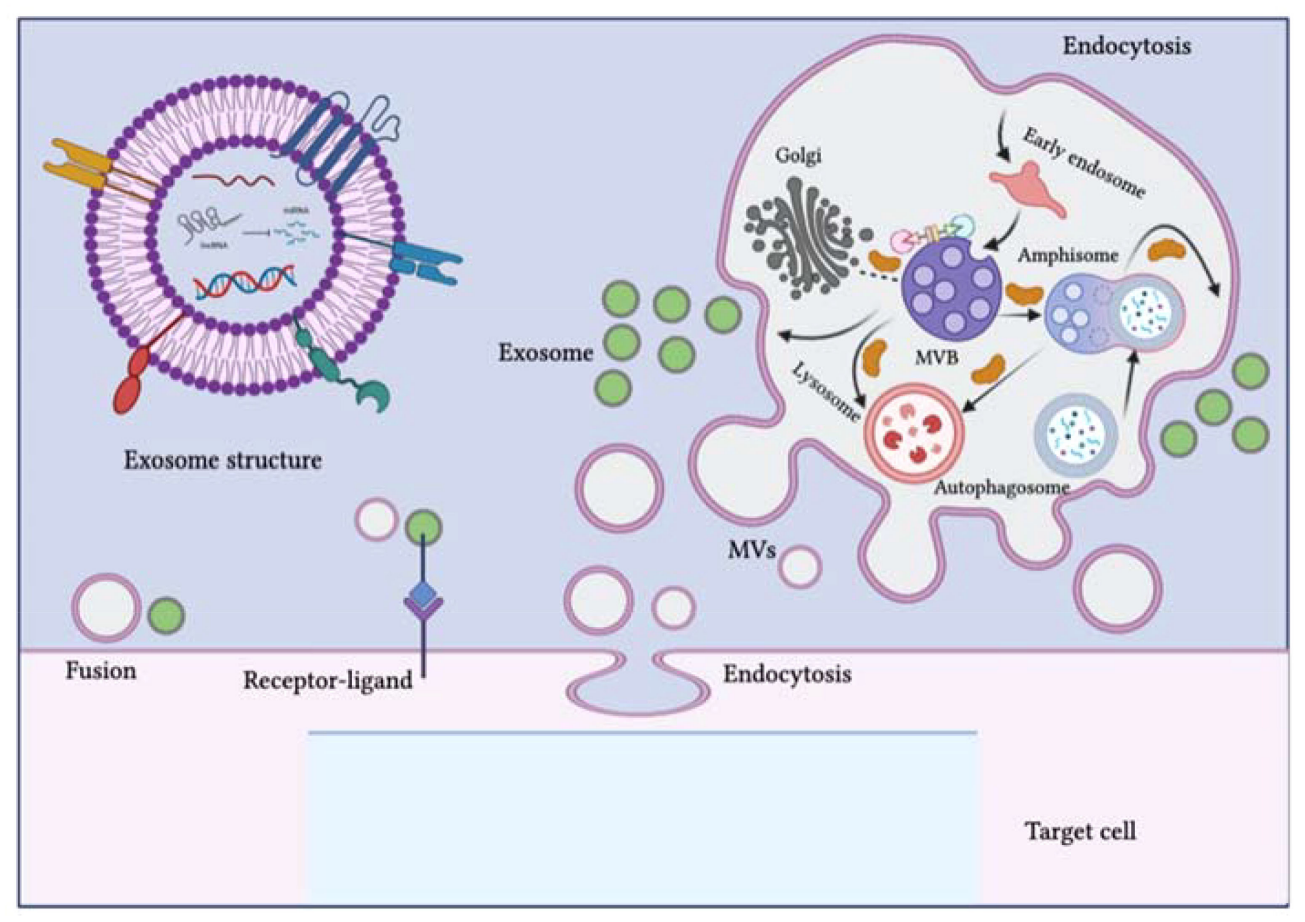
Schematic representation of the biogenesis of extracellular vesicles (EVs). EVs can be classified into three subclasses: exosomes, microvesicles (MVs) and apoptotic bodies. The biogenesis of exosomes is complex and occurs within multivesicular bodies (MVBs) located in the cytoplasm. Different molecules and ESCRT complexes participate in the loading, budding and maturation of exosomes in an ATP-dependent or independent manner. Exosomes are delivered from the Golgi apparatus, the endosomal pathway and the cytoplasm and intracellular trafficking within the endosomal compartment and exosomes is mediated by Rab proteins. MVBs can fuse with lysosomes, autophagosomes or plasma membranes. After fusion with the plasma membrane, exosomes are released into the extracellular environment. The biogenesis pathway of exosomes can interact with the autophagy flux, as seen with the development of hybrid bodies called amphisomes, which are formed by the fusion of MVBs with autophagosomes and eventually integrate with lysosomes or the plasma membrane. EVs can affect target cell signaling pathways and fate through three possible mechanisms: direct fusion with the plasma membrane, receptor-ligand interaction and endocytosis. The figure was designed using Adobe Illustrator.

Extracellular vesicles (EVs) are comprised of the lipid bilayer and are inherently secreted by different cells (**Table 1**; **Figure 2**) (11). These vesicles can be categorized into three subsets depending on their size i.e., (1) apoptotic vesicles (500-2000 nm) are secreted during planned cell death from the cell membrane, (2) microvesicles ranging in size (150-1000 nm) are also released from the cell membrane and (3) exosomes (30-150 nm) arise from endosomes (12). Exosomes are small-sized lipid bilayer extracellular vesicles (EVs) that are secreted following the integration of multivesicular bodies (MVBs) with cell membranes into the microenvironment (13). Exosomes are genetically produced by several cells including dendritic cells, neurons, T and B cells, epithelial cells, tumor cells and mast cells (14). Such types of vesicles were originally identified as tiny “cellular dust” squeezed out of cell storage which followed the pathway of cellular waste removal (15). Furthermore, earlier studies have shown that exosomes play a vital role in intracellular communication in various cellular processes, including antigen presentation, signal transduction, and immune feedback (16). Exosomes have become an increasingly effective and promising drug delivery carrier. They have comparatively little cytotoxic effect and immunogenicity because they are natural transporters for payloads such as lipids, proteins and nucleic acid and also, they have enhanced biocompatibility due to plasma membrane proteins like fibronectin and tetraspanin (17). Unlike LNPs, Exosomes have demonstrated high stability in the plasma due to their ability to circulate *in vivo* for an extended period (18). Exosomes also have the potential to cross the blood-brain barrier (BBB) by multiple pathways (19). In contrast, how to transport cargo to the CNS competently with LNPs has been consistently a resilient question. However, unmodified exosomes are most likely to be removed from circulation quickly after intravenous injection and are mainly dispersed to the lungs, liver, gastrointestinal tract and spleen (20, 21). This can be done by genetic engineering processes including roosting peptides or ligands and transfection on the membrane of exosomes and can be manifested in improving therapeutic and target activities (22, 23). This review briefly discusses the role of exosomes in HIV-1 vaccine development, immunological responses, their role as biomarkers and current challenges in the delivery system.

**Table 1:** Different types of extracellular vesicles.

EVs	Size	Markers	Biogenesis
Exosomes	30-150 nm	CD63, CD9, CD81, ALIX, TSG101	Generated by inward budding of the membrane of MVBs through ESCRT-dependent and ESCRT-independent and released into the ECM. Upon fusion of MVBs with the plasma membrane
Microvesicles	50-2000 nm	ARF6, VCAMP3, Selectins, Annexin A1, CD40	Pinching off from the membrane
Apoptotic bodies	500-5000 nm	TSP, C3b, Phosphatidylserine , Annexin V	Generated from apoptotic cells following stimulation of apoptosis-related pathways

## 2 Exosomes: development, configuration and function

### 2.1 Development of exosomes

Exosome production is initiated from the endosomal pathway, where premature endosomes develop into mature ones or multivesicular bodies (MVBs), and at the same time, the endosomal membrane integrates to form intraluminal vesicles (ILVs) within the organelle lumen (24). At first, the introversion of the cell membrane moves towards budding formation, followed by the incorporation of cell-surface proteins. This process involves the movement of fluids and extracellular contents. The budding procedure leads to the *de novo* production of initial endosomes or the integration of the premature endosomes with the components of the trans-Golgi network (TGN) and endoplasmic reticulum (ER). The endosomal pathway involves the development of premature endosomes into mature endosomes, which then grow into multivesicular bodies (MVBs) containing intraluminal vesicles (ILVs) through the inward folding of the endosomal limiting membrane. In this method, several proteins are arranged on budding endosomal membranes inwardly and a few cytosolic matters are encapsulated within the intraluminal vesicles. Multivesicular bodies will then select between two cell fates. Several MVBs are transferred to the cell membrane via the network of the cytoskeleton, where they secrete intraluminal vesicles in the extracellular environment which eventually develop into exosomes. Upon exocytosis, post-integration occurs with the plasma membrane. The other fate leads MVBs to deprivation in combination with autophagosomes or lysosomes (25, 26).

Various pathways for biogenesis have been investigated, including the pathway that depends on the endosomal sorting complex required for transport (ESCRT) which is commonly used. ESCRT-0 organizes payloads and the ESCRT-I–ESCRT-II complex stimulates membrane budding and internalization of cargo. As a result, ESCRT-III accounts for membrane scission (27). Once released, recipient cells might take exosomes in through several mechanisms, involving membrane fusion, receptor-mediated interactions and endocytosis (28). Exosomes are most probably target lysosomes, after they enter recipient cells, where they decompose their payloads (29). Cargo delivered by exosomes can also stimulate various pathological and physiological actions in recipient cells (30).

### 2.2 Configuration of exosomes

**Figure 3:**
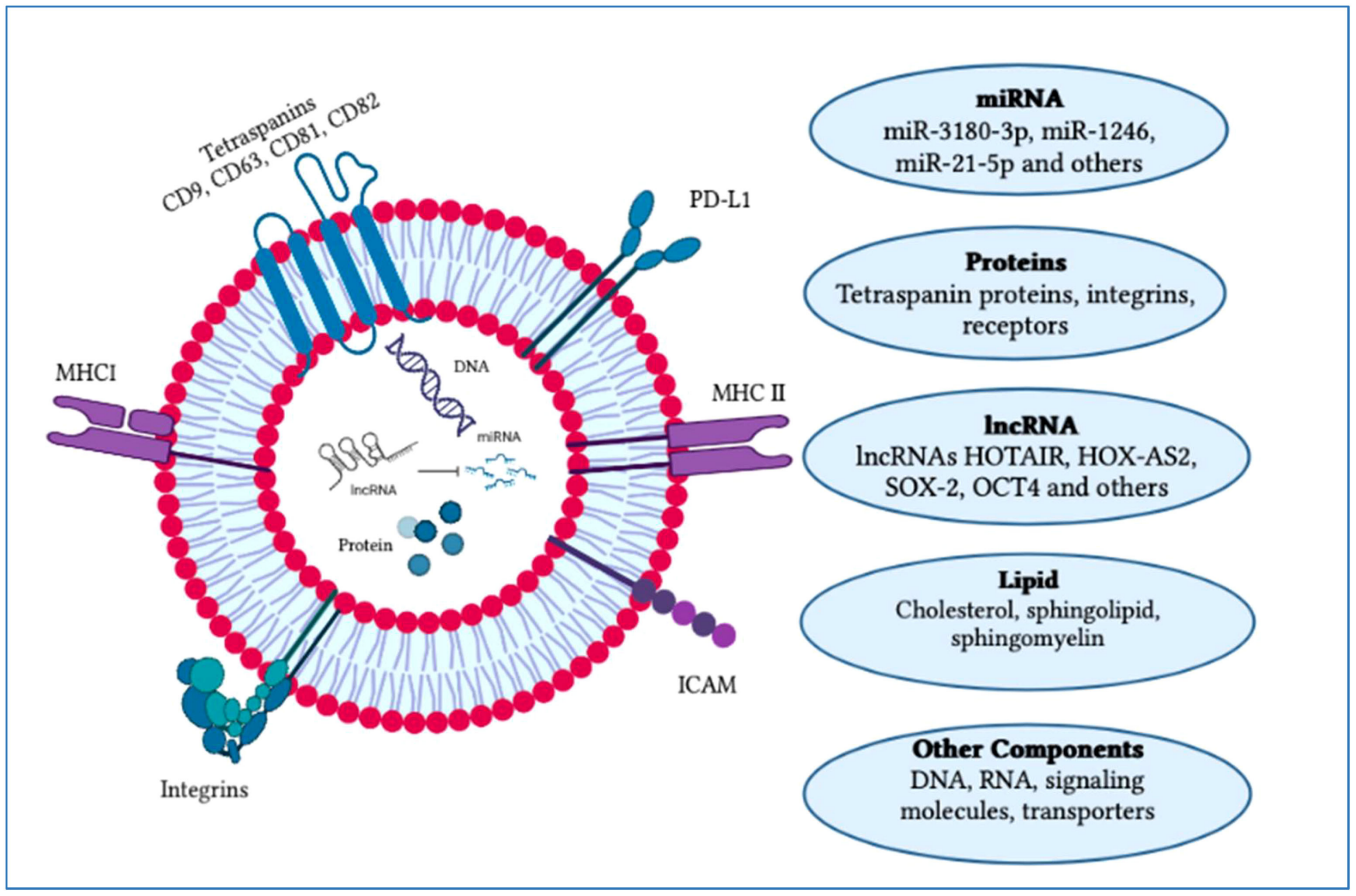
Typical structure of an exosome and its major components. An exosome is composed of an outer layer with a lipid bilayer that includes various functional components such as lipids, proteins, mRNA, miRNA, siRNA, lncRNA, signaling molecules and transporters. These exosomal constituents play a crucial role as potential biomarkers and in cancer progression. Currently, exosomes are being explored for their potential therapeutic applications and use in vaccine development research. The figure was created using Adobe Illustrator.

The factors that determine exosome yield and composition are environmental stimulation, parent cells and physiological conditions. Exosomes comprise a collection of proteins including extracellular matrix, membrane proteins and nuclear proteins. Cytosolic metabolites along with nucleic acids like DNA, non-coding RNA and mRNA can be observed in exosomes (31, 32). On the outer surface of exosomes, there is an attachment of the glycan canopy to the outer leaflet lipids and surface proteins (33). Under this glycan canopy, phospholipids, cholesterol, ceramides, diglycerides, glycerophospholipids and sphingolipids are abundantly found in exosomes as compared to the parent cells (34, 35). CD63 and HSP70 are well-known proteins that can be found in exosomes (36). Additionally, several tetraspanin proteins, such as CD81, CD82, CD37, CD9 and CD63, are highly abundant in exosomes (37). Besides these components such as proteins and lipids, some other components like functional RNA are also reported to be found in exosomes such as mRNAs, microRNAs, LncRNAs, non-coding RNAs and exospheres (**Figure 3**) (38).

### 2.3 Functions of exosome

Conventionally, there are various pathways for direct cell-to-cell interaction including cell surface protein communication or interaction with distant cells through released factors and gap junctions (39). Recent trends in exosomal studies suggest that lipids, proteins and nucleic acids that carry the exosome’s function can also stimulate cell-to-cell interactions (40). Exosomes carrying functional molecules might affect recipient cells through various pathways for example (1) direct activation of target cells through surface ligands, (2) transfer of activated receptors to the target cell and (3) genetic reprogramming of recipient cells through RNAs, lipids and protein delivery (41). Exosomes perform an important function in immunomodulation, such as antigen presentation, immune tolerance, immune suppression and immune activation. For instance, exosomes released from CD4+ and CD8+ T-cells have been shown to influence DCs, resulting in antigen-specific T-cell dormancy (42). Regulatory T-cells secrete exosomes which initiate immune suppression and Th1 immune feedback inhibition (43). CD73-expressing exosomes can also be released by regulatory T-cells, which might inhibit the stimulation of CD4+ T-cells (44–46). Different methods of exosome isolation which are currently employed are shown in **Table 2**.

**Table 2:** Different approaches of exosome isolations.

Isolation methods	Principle	Advantage	Drawbacks	Reference
Ultracentrifugation	Size, density and sedimentation properties	High purity	Low recovery, large demand for samples, long operation time	(47)
Immunoaffinity methods	Affinity purifications	High purity and specificity	High cost, restriction in the number of samples	(48)
Size-exclusion Chromatography	Size	Easy operation, low cost	Low specificity and purity	(49)
Polymer precipitation	Polymer precipitation	Easy and fast operation	Low recovery, low purity	(50)
Microfluidics-based methods	Size and density, or chemical properties	High efficiency, high purity, fast operation	Complexity of device	(51)

## 3 Exosomes‐based vaccines

Tumor cell-associated exosomes have been used as therapeutic agents in quick antitumor immune responses. Moreover, vaccines based on exosomes presented beneficial results against various types of infectious diseases. Exosomes isolated from native tumors and immune cells can be used as tumor vaccines (52). Various active components on exosomes such as costimulatory molecules and MHC assist immune cells antitumor responses. Emerging studies in summarizing exosome cargo have also depicted the gradual increase of active molecules that might be adapted in tumor immunotherapies (53). Many preclinical studies have been conducted on exosomes, as there has been extensive investigation into their potential function in tumor immunotherapy (54). In glioblastoma-infected individuals who obtain anti-survivin immunotherapy, the lower levels of CD9 +/SVN + and CD9 +/GFAP +/SVN + exosomes have been associated with the sustained survival of patients (55). Furthermore, exosomes isolated from tumor cells have DNA threads, which can produce immune feedback by following the STING/cGAS mechanism and as a result, exosomes might play a significant role in cancer immunity control, particularly in the context of immunotherapy checkpoints (56). Dendritic cell-based exosomes sourced from individuals with tumor afflictions have shown potential for use in immunotherapy, as some early experimental trials have found them to be safe and effective. Immunotherapy associated with DCs-derived exosomes has lower costs, more bioavailability and biostability (57). In an experiment, the researcher vaccinated patients through the administration of autologous DCs exosomes against metastatic melanoma and revealed the safety of using them in a first phase 1 clinical trial (NCT01159288). However, the authors did not find profound CD4+ or CD8+T-cell feedback and it is still needed to study the pathway followed by the vaccine antigen delivery (58). Moreover, in a different experiment involving an individual with NSCLC, dendritic cell-based exosomes were found to enhance natural killer cell activity and elicit MAGE-specific T-cell responses (59). In the phase II clinical study of immunotherapy that involved the administration of IFNγ DCs-exosomes, researchers aimed to enhance the NK and T-cell immune responses in individuals with ongoing NSCLC. Authors demonstrated that DC exosomes might enhance the NK cells antitumor immunity in individuals. Though, the major clinical report, a 50% progression-free survival (PFS), might not be attained and non-reliable immune feedback was demonstrated by that study (60). The final achievable choice is to associate the dendritic cells-derived exosome immunotherapy with natural killer-derived therapies for inducing synergistic immunogenic results against tumors dependent on NK cells (61). Furthermore, it has been seen that the organization of an immortalized DC line library, which is regulated to accelerate only one MHC II component and no MHC proteins, might make continuous construction of promising DCs-based exosomes resulting in fewer treatment costs and periods of cell culture. DCs-derived exosomes have been revealed to have significant potential in treating glioblastoma in mouse models, suggesting that this therapy could be a promising therapy against glioma because brain tumor cells are resilient towards immune cell recruitment (62). Reports based on current investigations and clinical trials on exosomes indicated that exosomes can become immunotherapeutic agents against several tumor cell lines. Autologous DCs-exosomes are administered in both clinical trials, following the current GMP criteria. While, current clinical trials are significant in demonstrating the feasibility as well as short-term safety of administering autologous exosomes, safety intentions for treatments based on exogenous exosomes-derived products will undoubtedly require more rigorous evaluation. Although a few experiments have reported methodology for high exosome yield and advancement in biocompatibility, for confirmation there is a need for more clinical and preclinical trials (63). After all, a relatively unified form and smaller size permit exosomes to effectively discharge by the mononuclear phagocyte system, elongating their time for circulation along with depicting their advantage in cell-to-cell interaction.

Mucous membranes are the routes through which HIV enters the body. Immature dendritic cells (iDCs), which serve as sentinels and detect alien pathogens, are the first cells it comes into contact with. The pathogen is endocytosed by iDCs when they come into direct contact with it, which causes iDC activation and migration to secondary lymphoid organs. In the lymph nodes, mature dendritic cells (mDCs) prime CD4+ and CD8+ T lymphocytes (CD4TL, CD8TL) for the active immune response by presenting them with epitopes generated from internalized viruses (64). However, the presentation of the HIV particle to CD4TL by an iDC doesn’t lead to their activation of an immune response (65). Instead, a phenomenon known as trans-infection occurs often referred to as the “Trojan horse” hypothesis (4). It is important to note, however, that the HIV particle’s presence within the iDC leads to its rapid degradation. Hence, it is plausible that the infection of CD4TL happens via two distinct pathways: trans-infection as well as through the iDC’s production and release of newly formed viral particles resulting from the infection (64). Dendritic cell immunotherapy (DC-IT) is one of the most promising new HIV treatment approaches. Therefore, we must keep track of the complex biomarkers and signaling processes related to HIV infection and DC operation. Exosome secretion is one such method and those produced by DCs have been found to control almost every component of the immune system. DC-exosomes may include major histocompatibility II complexes, which enable them to function as antigen-presenting entities and activate the adaptive immune response, including both CD4TL and CD8TL, depending on the maturity state of the parent DC (66).

In a recent edition of medical hypothesis, Ellwanger and colleagues presented their hypothesis, drawing on initial data gathered by Pontillo et al. which explored exosome secretion and CD4TL response to DC-IT (67). The researchers examined the expression of 84 genes implicated in the anti-HIV response as well as the expression of the TSG101 gene, an exosomal marker, using monocytes and monocyte-derived DCs obtained from six phenotypically similar patients engaged in a DC-IT experiment. Based on whether the genes were primarily upregulated (group B) or primarily downregulated (group A) in comparison to control monocytes, Pontillo et al. categorized the patients into these two groups. Despite the comparable phenotypes of the patients’ disorders, group A showed elevated levels of CD4TL than group B, suggesting that these individuals would be more receptive to DC-IT. Additionally, they discovered a negative correlation between TSG101 expression and anti-HIV response genes (group A showed an increase while group B showed a reduction). This would suggest that exosome synthesis and secretion affect HIV response (67, 68). These first results highlight intriguing features of HIV exosome signaling. Through antigen presentation and secondary immune response activation, exosomes are thought to often assist the immune response. The fact that TSG101 was downregulated while CD4TL was elevated suggests that exosomes may exert the opposite impact on HIV and DC-IT (67).

Recent research suggests that although exosomes and HIV use similar cell pathways, they are distinct particles. Subra et al. used immunocapture and ultracentrifugation to effectively remove HIV particles from exosomes. They discovered that DC-exosomes caused CD4TL to undergo apoptosis and could not propagate HIV infection after pulsing DCs with the virus. Apoptosis was not triggered by HIV particles released by DCs and isolated from exosomes (68). The dependency of DC exosome release on dendritic cell immunoreceptor (DCIR), the same receptor used by DCs and CD4TL to bind the HIV virion, was discovered in a recent work from the same research group by Mfunyi et al. (69). Exosome production was inhibited by the inhibition of this receptor, highlighting the similarities between the biological processes exploited by HIV and exosomes (69). Further verification of these findings that build off the data presented by different researchers will provide significant insights into exosome and HIV biogenesis, immunity, and treatment.

## 4 Exosome-based infectious diseases vaccine

Vaccine development for infectious diseases is considered one of the most advanced areas in gene therapy. Currently, major studies utilize mRNA as a therapeutic agent to develop RNA vaccines. Target cells have taken up mRNA vaccines followed by translation into antigens to produce immune responses. The major factor in delivering mRNA is to organize a reliable and efficient delivery system. Therefore, many mRNA vaccines developed for clinical trials or research employ virus-like particles (VLP) and lipid nanoparticles (LNP) as delivery systems (70). However, these vesicles are associated with issues like immunogenicity and cytotoxicity (71). Exosomes are considered to have the potential to surpass these drawbacks.

Adding up more analysis has suggested that exosomes have an important function in cell-to-cell regulation and interaction. Exosomes are thought to take part in the pathogenesis of several retroviral infections (**Table 3**). Antigens can be transferred through integration into the host cell’s exosomes or by bacteria, parasites and viruses (95). During the infection pathway, exosomes can transmit pathogenic agents such as proteins, carbohydrates, lipids and nucleic acids which produce host defense response and immunity or stimulate immune evasion. Exosomes loaded with pathogens can perform the function of antigen vehicles that might produce acquired immune feedback. They can initiate T-cell stimulation directly by transmitting costimulatory molecules, modified antigens and MHC molecules (96). These pathogens-loaded exosomes can also deprive immune responses by following several pathways. For example, exosomes loaded with microorganisms like HIV, leishmania gp63 or Nef can suppress T-cell regulation leading towards apoptosis in immune effector cells (97). Moreover, exosomes isolated from infected cells might increase infection in normal cells (98). The significant role played by exosomes in the progression of infection provides new opportunities to treat infectious diseases. The use of exosomes as compared to other nanocarriers in the treatment of infectious diseases has several benefits such as (1) regulated bio-delivery because of their capability to circulate in the whole body; (2) a steady environment for a therapeutic agent like RNA; (3) effective cellular uptake by APCs; (4) transported antigen has a natural function among cells.

**Table 3:** Exosomal cargoes in retroviral infection.

Exosome cargo	Target cell	Origin of exosome	Function	Reference
TAR	293 T-cells	HIV-infected cells	Protecting cells from apoptosis caused by viruses in infected cells	(72)
	Primary macrophages or mouse neuronal cells	HIV-infected cells	Stimulating the secretion of IL-6 and TNF-α, the pro-inflammatory cytokines	(73)
	HSC3 HNSCC and H1299 lung cancer cells	HIV-infected cells	Development of lung cancer and HNSCC	(74, 75)
vmiR88 and vmiR99	THP-1 macrophages	IV-infected macrophages and the sera of HIV + individuals	Inducing an inflammatory response	(76)
miRNA-29b	Neurons	Astrocytes treated with both morphine and HIV Tat	Down-regulation of PDGF-B expression and neuronal cell death	(77)
miRNA-155-5p	Cervical cancer cells	HIV-infected T cells	Up-regulating the expression of cytokines such as IL-1, IL-6, and IL-8	(78)
Tax and HBZ	PBMCs	HTLV-1-infected cells	Infection progression	(79)
Nef	T-cells	HIV-1 infected cells	Making the latent cells more vulnerable to HIV infection	(80)
	T-cells	T-cells	inhibiting the generation of CD4+ EVs from T-cells	(81)
	T-cells	Macrophages	Inhibiting T-cell function via beta-COP-dependent pathway and degradation of MHC-I and CD4+ molecules	(82)
	B cells	HIV-1-infected macrophages	Inhibiting the adaptive immune response by deterring the IgA and IgG production in B lymphocytes	(83)
	Macrophages	HIV-1 infected cells	Increasing secretion of pro-inflammatory cytokines	(84)
	SH-SY5Y neuroblastoma cells	Plasma from patients with HAD	HAND progression	(85)
	Microglia	HIV-infected microglia	HIV-induced neuropathogenesis	(86)
CCR5	CCR5-null cells	PBMNCs and CCR5 + ovary cells	Enhancing HIV-1 infection	(87)
CXCR4	CXCR4-null cell	Megakaryocytes and platelets	Enhancing HIV-1 infection	(88)
APOBEC3G cGAMP	PBMCs	HIV-infected cells	Preventing virus production	(89)
cGAMP	DCs	HIV-infected cells	Interferon upregulation	(90)
Tat and TAR	Not assigned	CSF samples of HIV-positive individuals	Inducing pro-inflammatory responses	(91)
Tat	SHSY-5Y	Tat-expressing primary astrocytes	Neurite reduction and neuron death	(92)
Tax	Microglia cells	HTLV-1-infected cells	Inducing the production of proinflammatory cytokines	(93)
	Uninfected PBMCs	HAM/TSP patient’s PBMCs and CSF samples	Lessening the CD4 + CD25 + T-cells	(94)

The rapid development of the mRNA vaccine was catalyzed by the COVID-19 outbreak. With the approval of the Pfizer-BioNTech vaccine BNT162b2 and the Moderna vaccine mRNA-1273 for human use, researchers have recognized the importance of mRNA vaccines and there are increasing trends in the advancement of new mRNA vaccines (99). However, due to the unknown mechanism of action and potential to act as a therapeutic agent, there is still a limited number of research that has been conducted on exosomes as RNA carriers to treat infectious diseases as compared to other nanocarriers. Many investigations have used exosomes to develop an mRNA vaccine for the COVID-19 outbreak. For instance, Tsai et al. loaded exosomes isolated from 293F cells with SARS-CoV-2 spike encoding mRNA and nucleocapsid proteins. The outcomes suggested that exosomes loaded with mRNA can produce cellular as well as humoral immune responses. Simultaneously, they also compared the delivery efficiency of exosomes and LNPs by delivering Antares2 mRNA into both carriers and investigating the luciferase efficiency of each group after co-incubation with human cells. The results indicated that mRNA-loaded exosomes induced higher expression levels than LNPs (100). Although several studies have investigated the use of exosomes for pathogen delivery, including proteins to treat infectious diseases, there is still a need to use exosomes loaded with mRNA. However, various studies have reported the feasibility and promising potential of RNA-loaded exosomes for infectious disease therapy, but more investigations are required to enhance the knowledge in this area.

## 5 HIV and exosomes

**Figure 4:**
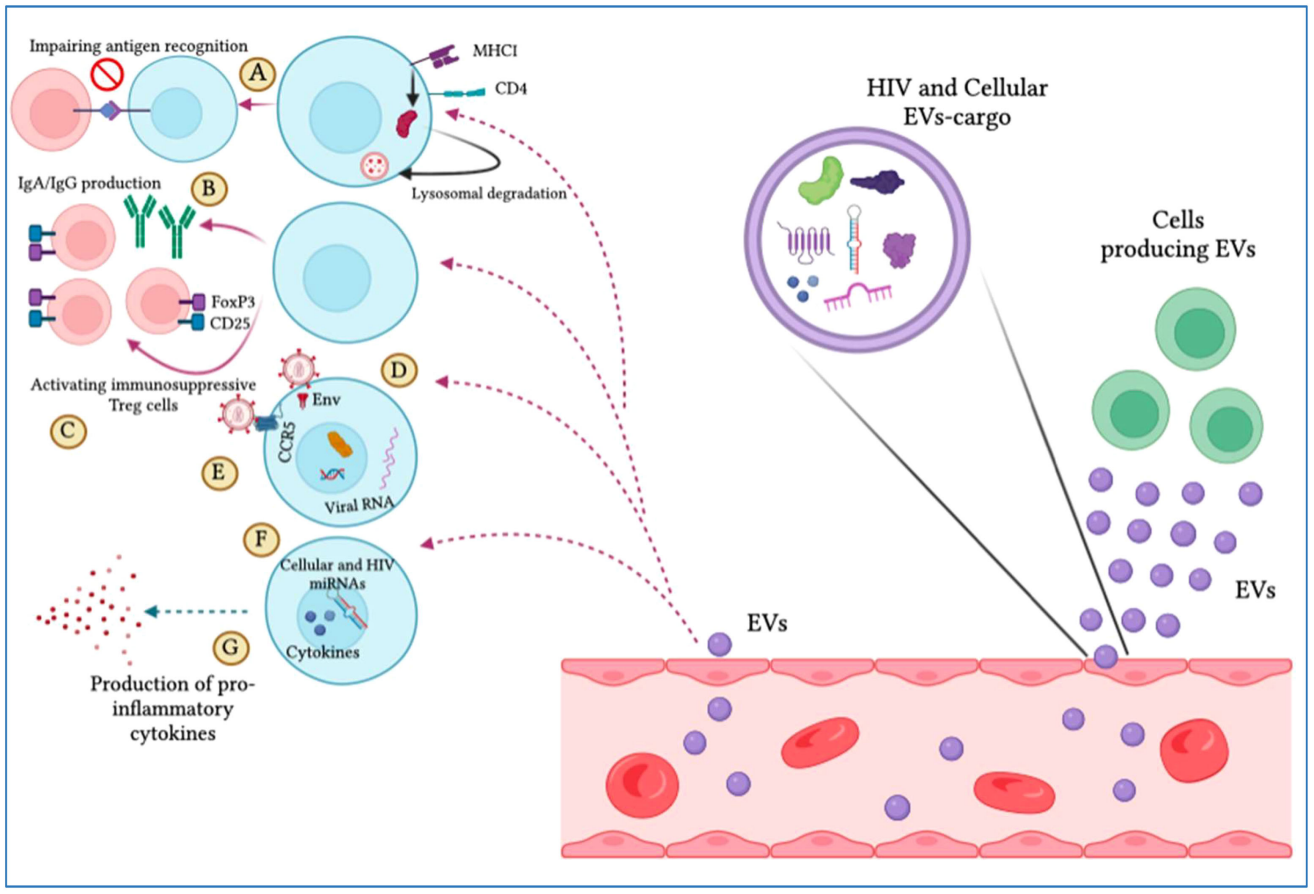
Extracellular vesicles (EVs) display pro-HIV actions. The figure illustrates that different cells produce EVs that are released into the blood circulation, expressing cellular and viral particles that induce pro-viral ramifications on target cells. These include **(A)** hindering antigen identification by MHC-I and CD4 lysosomal degradation, **(B)** impeding IgA and IgG production by B cells, **(C)** complement immunosuppressive T regulatory cells, **(D)** increasing viral infection by integrating with target cells through the envelope protein, **(E)** promoting viral tropism mitigation, **(F)** stimulating the viral promoter to provoke HIV replication, and **(G)** triggering the production of pro-inflammatory cytokines.

As described earlier, EVs can stimulate HIV infection (**Table 4**; **Figure 4**). Exosomes can be isolated from HIV-1-infected cell cultures and the blood serum of infected patients (120). While exosomes from latent HIV-1-infected Jurkat cells (J1.1) contain some viral proteins, such as the precursor form of Env protein (p160) and Gag protein, they do not contain intact HIV-1 viral molecules (72). However, the precursor of various HIV-encoded mRNAs, known as HIV-TAR RNA, can be found in exosomes from infected individuals and may assist in the attachment of the viral Tat protein, leading to increased HIV replication and transcription (121). When exosomes loaded with TAR-RNA are introduced to primary macrophages, they can induce the production of tumor necrosis factor-b (TNF-b) and interleukin-6 (IL-6) cytokines (120). Recently, the use of exosomes along with other EVs is an emerging tool to diagnose and treat HIV-1 disease (122). Finding out the possible association between HIV and the secretion procedure of exosomes might facilitate the treatment of HIV-1 infection and its derived complications, which leads to the generation of novel therapeutic agents and medications (123). Besides all the efforts in the last two decades to discover an efficient and compelling vaccine to defend the immune system from the attack of HIV-1 infection, there is still a requirement to solve issues relating to the mechanism associated with this therapy (124). Addressing these issues might involve the determination of an unknown pathway followed by host-pathogen communication and the capability of the virus to escape the immune system. For example, one of the mechanisms to evade the immune system is to take advantage of intracellular interaction procedures. Cytoplasmic channels, such as cell-cell tunnels or tunneling nanotubes develop conditions that facilitate evasion of immune detection. Through these components, cellular particles and macromolecules can be physically transferred from one cell to another cell. These cytoplasmic channels could be utilized in the proliferation of microbes when two immune cells make synapses to show antigens in humoral immunity. In this way, the virus could transpose from one T-cell to affect all of them (125).

**Table 4:** Exosomal cargoes released from HIV-infected cells.

Type of cargo	Exosome source	Expression	Reference
Protein			
Tat	Engineered human cellular exosomes to express HIV Tat	Up-regulation	(101)
Nef	Expression of HIV Nef proteins in HEK 293 cells	Up-regulation	(102)
Hck and ADAM17	Plasma, myeloid cells	Up-regulation	(103)
Nef and ADAM17	Plasma	Up-regulation	(69)
Nef	pro-inflammatory vesicles	Up-regulation	(104)
DAP-3	Dendritic cells	Up-regulation	(105)
Nef	A3.01 T-cell line	Up-regulation	(106)
Nef-ADAM17-TNF-a	U937 cells	Up-regulation	(106)
Nef	Astrocyte cultures	Up-regulation	(107)
Fibronectin and galectin-3	HIV-infected DC	Up-regulation	(108)
Nef	HEK 293 cells	Up-regulation	(85)
Nef	Plasma, neuroblastoma cell line SH-SY5Y	Up-regulation	(109)
Nef domain		Up-regulation	(110)
Nef		Up-regulation	(111)
Nef	PBLs	Up-regulation	(112)
Gag	Jurkat T-cells	Up-regulation	(86)
Nef	Microglial cells	Up-regulation	(92)
Tat	Tat-expressing primary astrocytes	Up-regulation	(113)
**MicroRNA**		Up-regulation	
miR-155	Plasma	Up-regulation	(114)
miR-223	Plasma	Up-regulation	(114)
miR-548a	HIV-infected macrophage	Up-regulation	(115)
miR-30e	HIV-infected macrophage	Up-regulation	(115)
miR-338	HIV-infected macrophage	Up-regulation	(115)
miR-454	HIV-infected macrophage	Up-regulation	(115)
miR-518f	HIV-infected macrophage	Up-regulation	(115)
miR-1243	HIV-infected macrophage	Up-regulation	(115)
miR-1247a	HIV-infected macrophage	Up-regulation	(115)
miR-150	HIV-infected macrophage	Up-regulation	(115)
miR-29a	HIV-infected macrophage	Up-regulation	(115)
miR-302c	HIV-infected macrophage	Up-regulation	(115)
miR-636	HIV-infected macrophage	Up-regulation	(115)
miR-872	HIV-infected macrophage	Up-regulation	(115)
miR-875	HIV-infected macrophage	Up-regulation	(115)
vmiR-88	Serum	Up-regulation	(116)
vmiR-99	Serum	Up-regulation	(116)
miR-29b	–	Up-regulation	(77)
TAR miRNA	PBMC	Up-regulation	(72)
miR-130a	HIV-infected and cocaine-treated, human monocyte-derived macrophages	Up-regulation	(117)
miR-17	Human monocytic U937 cells	Up-regulation	(118)
miR-382	Human monocytic U937 cells	Down-regulation	(118)
RNA			
TAR RNA and TAR-gag	Jurkat E4 cells (HIV- infected T-cells) and other cell lines	Up-regulation	(72)
Nef messenger RNA	SH-SY5Y and HEK 293 cells	Up-regulation	(85)
AATK, SLC27A1 and CDKAL	Nef-expressing U937 monocytic cells	Up-regulation	(119)
MECP2, HMOX1, AARSD1 and ATF2	Nef-expressing U937 monocytic cells	Up-regulation	(119)
Nef messenger RNA	Neuroblastoma cell line SH-SY5Y	Up-regulation	(73)
Cytokine/chemokine			
Nef, IL-1, IL-2, IL-2Ra,	Plasma	Up-regulation	(24)

The complications related to the study of HIV-infected cell-secreting exosomes are due to specific features of HIV-1 viruses and exosomes. Their uptake pathways, biogenesis and biophysical molecular properties are distinctive and complicated. The size of the HIV-1 virus ranges from 100 to 120 nm which is slightly wider than exosomes with a diameter of 40-100 nm (126). The budding of HIV-1 has been proposed to contain several cellular procedures, for instance, those involving ALG-2 communicating protein X and gene 101 which is tumor-vulnerable, and also takes part in the biogenesis of exosomes (109, 127). The conjunction of exosomes and HIV-1 biogenesis indicates that HIV-1 molecules like RNAs and proteins, could be encapsulated in exosomes. However, the controversy surrounding exosomes purified from sera of HIV-1 infected patients raises the question of whether this could be a result of contamination. Though, intense purification methodologies comprising density gradient centrifugation, and immune-affinity methodologies can isolate exosomes from the HIV-1 virus (128). The significant resemblance between the exosome’s biogenesis and that of encased viruses especially HIV-1 leads to the ‘Trojan exosome’ theory of HIV assembly and cell-to-cell communication. This supports the hypothesis that HIV-1 may have evolved to utilize exosome biogenesis and cellular uptake mechanisms, enabling high-level-independent viral entry to the host cell (129). HIV components may be encased into intraluminal vesicles (ILVs), but there is uncertainty as to whether the mature progeny virus infects the cells or not (130). It might be possible that exosomes that are attached to the plasma membrane through their unique receptors on the cell surface or by the meeting of integral viruses might be responsible for viral infections. On the other hand, HIV might engage exosomes to enhance infection and evade the immune system. This method through which exosomes could assist viral contact with cells of adaptive and innate immune defense is very significant. It might be a pathway by which HIV acquires immunity against some neutralizing antibodies (131).

The constituents that comprise exosomes isolated from biological fluids are rigorously variable and it has been suggested that these constituents may be responsible for their biological activities. Current evidence suggests that HIV pathogenesis affected by exosomes might depend on their cellular source (132). Based on all of these circumstances, it has been found that exosomes purified from HIV-infected cells are more contagious and promote disease when compared with exosomes isolated from non-infected cells (133). Encountering the source of exosome secretion, it has been seen that the exosomal origin determines its effects on HIV (132). For instance, exosomes isolated from the blood serum of an HIV-infected individual may be extracted from different types of cells, including both uninfected cells and those infected with HIV infection. It has been proposed that HIV arrests the mechanism of exosome biogenesis that normally facilitates the transfer of different viral RNAs and proteins during viral dissemination (112). It has also been seen that HIV-infected cells-derived exosomes have components that can produce apoptosis and change the immune response. In this way, Lenassi et al. found that Nef exosomes produce apoptosis in peripheral blood lymphocytes of HIV-1-infected patients (101). Moreover, it was determined that TAR miRNA might downregulate cell death by 11 (Bim) protein which is a Bcl-2-like protein. These findings prompted Narayanan et al. to evaluate exosomes in PBMCs from both uninfected individuals and those infected with HIV-1 who were either long-term non-progressors (LTNPs) or receiving highly active antiretroviral therapy (HAART) (72). In another study, Lee et al. isolated exosomes from myeloid cells of HIV-infected individuals and found that they contained hemopoietic cell kinase (Hck), myeloid tyrosine kinase, and Nef. These proteins can lead to the secretion of pro-inflammatory cytokines and are associated with immune dysfunction in chronic HIV disease (134).

The EVs purified from human semen are shown to have the potential for antiretroviral effects. Madison et al. described the capability of EVs obtained from the semen of a healthy individual to suppress the replication of the virus when mixed with several HIV strains (135). Scientists debated that EV-stimulated interruption in the reverse transcript process causes disturbance in the replication of the virus. Interestingly, it has also been found that this antiretroviral activity was only shown by EVs obtained from semen but EVs derived from blood do not show this potential (135). The researchers found that EVs extracted from the semen of a healthy man can inhibit HIV infection spreading to vaginal cells and suppress replication of the virus in vaginal epithelial cell lines (136). These findings demonstrate the potential significance of investigating the antiretroviral properties of exosomes and other vesicles isolated from semen for the development of novel therapies to treat HIV-1 infection. Furthermore, investigations into the extraction of EVs from breast milk gave attractive features related to HIV inhibition. Results showed that EVs obtained from the breast milk of a female showed immunoregulatory functions (137). Naslund et al. described that EVs isolated from the breast milk of healthy donors inhibit HIV infectivity of MDDCs and transmission of the virus to CD4 T-cells (76). In conclusion, the effect of exosomes on HIV infection is still a matter of research. Generally, exosomes may regulate immune responses and can disrupt the pathogenesis of HIV, playing a related function in the pathophysiology of HIV. This response is thought to be stimulated by the exosomal load, which constitutes majorly non-coding RNAs and plasma membrane proteins.

### 5.1 Role of exosomes in HIV‐1 stimulated pro‐inflammatory response

Inflammation is a fundamental aspect associated with HIV infection. Experiments have shown that proteins from HIV infection can stimulate pro-inflammatory cytokine signaling mechanisms. Pro-inflammatory cytokines play a significant role in the pathogenesis of HIV, as they act as key regulators in the lifecycle of HIV infection and the apoptosis of T-cells (91). It has been shown that exosomes transmit viral components and pro-inflammatory cytokines. For instance, exosomes obtained from CSF specimens of HIV-infected patients possess TAR and Tat proteins (73). These viral particles functioned as a vital inducer of pro-inflammatory responses (84). Mukhamedova et al. demonstrated that exosomes containing Nef might be engulfed by macrophages to enhance pro-inflammatory cytokines release via stimulation of NLRP3 inflammasome and ERK1/2 phosphorylation (138). Moreover, Chettimada and coworkers investigated the exosomal protein cargo from plasma purified from ART-treated HIV-infected individuals and healthy controls. In HIV-infected individuals, plasma exosomes accompanied by enhanced oxidative stress markers and Notch4 were increased. Activation of pro-inflammatory feedback during the pathogenesis of HIV was observed when THP-1 monocytes were treated with these exosomes, leading to overexpression of genes responsible for interferon responses (116). Sampey et al. reported that besides protein payloads, exosomes secreted by HIV-1 infected patients have plenty of TAR miRNAs and HIV-1 TAR-RNA, which might have a significant function in the pathological procedures related to HIV infection. They described that co-culturing of exosomes obtained from the cells of HIV+ individuals with key macrophages or neuronal cells of mice activates the release of TNF-α and IL-4, the pro-inflammatory cytokines by stimulating NF-κB constituents through attachment to PKR and potentially to TLRs (84). Moreover, in addition to this vmiR99, vmiR-TAR and HIV vmiR88 are also found in exosomes released from the blood serum of HIV-infected patients and macrophages associated with HIV. As ligands of the TLR8 signaling pathway, these miRNAs generate an inflammatory response and function as chronic immune mediators (107). Kulkarni et al. described that aside from viral cargoes, HIV-1 infected DCs derived exosomes comprised galectin-3 and fibronectin, which functions in up-lifting the expression of pro-inflammatory cytokines containing TNF-α, IFN-γ, RANTES and IL-1β, along with stimulation of p38/stat mechanism in T-cells (78). These findings demonstrate that exosomes derived from infected cells can trigger inflammatory feedback.

### 5.2 Role of exosomal cytokines and chemokines in HIV-1 infection

Delivery of cytokines by microvesicles and exosomes triggers the signaling role of these molecules. In a recent analysis, Konadu et al. extracted exosomes/microvesicles that comprise 21 cytokines and chemokines from plasma and performed a comparison of their ratio among infected and uninfected individuals. The researchers evaluated the ratio of all chemokines and cytokines present in measured isolated fractions of exosomes which showed a significant increase in patients than in healthy individuals (139). Mainly, the correlation of most of these chemokines and cytokines with exosomes/MVs is still deficient for approval by mass spectrometry or other protein-authenticating methodologies. Therefore, the increased ratio of these crucial immune components in the EV fraction of HIV-positive individuals highlights the significant capability of exosomes as delivery pathways for addressing HIV infection and other pathological conditions, which should be further investigated in upcoming research.

## 6 Role of exosomes as HIV‐1 biomarkers

Identification of particular exosomal RNAs or proteins in EVs of plasma isolated from HIV-infected individuals might facilitate HIV infection diagnostic purposes (140). In this regard, the estimation of specific miRNA in EVs has substantial potential. Exosomes isolated from a subclass of HIV-1+ controllers revealed that miRNA-21 was down-regulated in HIV-controlled individuals, in decreasing order with CD4 T lymphocytes. As a result, researchers proposed exosome-based miRNA-21 as a promising and valuable prognostic biomarker in HIV-1-infected elite controller patients (141). Aβ accumulation, demonstrated by HNAD, is a traditional biomarker for Alzheimer’s disease and is particularly prevalent in susceptible individuals (142). Sun et al. reported that neuron-isolated exosomes purified from HIV-infected and non-infected individuals suggested that neuropsychiatric disorders reduce the total number of neuron-based exosomes and extend them with the help of neurofilament-light (NF-L), Aβ, and high-mobility group box 1 (HMGB1). These studies indicate the potential role of neuron-based exosomes in the development of cognitive impact biomarkers in HIV+ patients (143).

EVs are also promising agents as biomarkers for HIV-infected drug addicts (144). To examine the impact of tobacco and alcohol, which are common in HIV patients, on the chemokines and cytokines of exosomes, Kodidela et al. investigated the levels of chemokines and cytokines in the plasma exosomes and plasma of different groups of HIV patients (145). They proposed that IL-8 levels were elevated in individuals with HIV who consumed alcohol, while IL-10 levels were higher in HIV-infected individuals who smoked, in comparison to HIV-positive non-drinkers and non-smokers. The outcomes indicated that the use of tobacco and alcohol can alter the chemokines and cytokines pattern of exosomes and may play a critical role in disease propagation and toxicity in HIV-infected drug addicts (145).

Finally, another study conducted a proteomic analysis of plasma exosomes isolated from HIV+ patients who consume tobacco, alcohol, or both daily. They examined the levels of various proteins, including alpha-2-macroglobulin, properdin, and hemopexin, in exosomes extracted from HIV+ smokers and drinker groups. These proteins in exosomes may be altered and could serve as potential biomarkers for drug abuse in individuals with HIV (146).

## 7 Exosome-based HIV-1 vaccine development and therapy

Despite the benefits associated with ART therapy in modifying the quality of HIV+ individual life, there are various risks and rigorous side effects related to this combination of treatment, so advancement in discovering alternative therapeutic agents or effective vaccines is essential for HIV-1-infected individuals (147). Almost all living organisms, such as bacteria and viruses, excrete exosomes in extracellular media. As previously highlighted, exosomes transmit big proteins, nucleic acid and lipids through their cargo.

**Figure 5:**
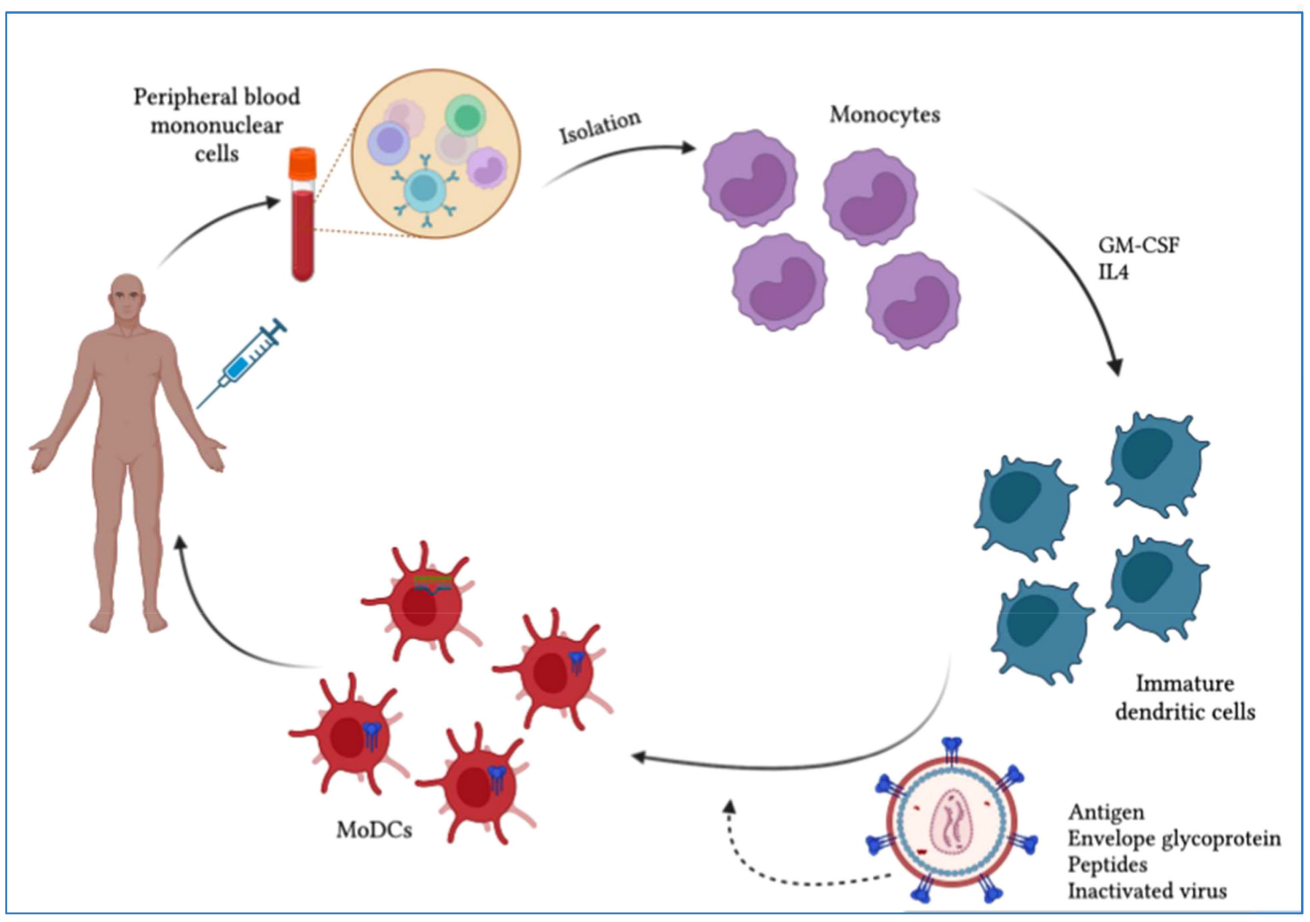
Scheme of dendritic cell (DC)-based therapeutic vaccine development. Monocytes are purified from PBMCs obtained by leukapheresis from the patient for the development of autologous DC vaccines. To induce immature dendritic cells (iDCs), monocytes are stimulated with IL-4 and GM-CSF. These iDCs are then loaded with HIV-1-derived antigens and allowed to mature into antigen-presenting dendritic cells. The resulting mature DCs can be used to develop a vaccine that is administered to patients to elicit a specific T-cell response to the HIV-1 antigen.

Some molecules such as proteins, nucleic acid-related signal transducers, lipids, membrane trafficking, T-cell stimulators and anti-apoptotic particles present on the surface of exosomes also showed immune regulatory functions. Cholesterol (CE), triacylglycerol (TAG) and cardiolipin also constitute exosomes. Triacylglycerol and cholesterol are located in the core of lipid droplets whereas cardiolipin is abundant in mitochondria (148). Ubiquitous and cell-specific proteins are both expressed selectively in exosomes from innate cells. In chronic inflammatory procedures, exosomes perform a vital role. For instance, exosomes derived from dendritic cells act as antigen-presenting components and may prolong the response of Th2 cells to dendritic cells to modulate inflammation and immunity (**Figure 5**) (149). Exosomes derived from B-cells and mast cells also drive responses of Th2 cells and enhance the Th2 environment (150). As biological nano-vesicles have a poor immunogenic profile, exosomes have high potential in the development of a new vaccine based on nanoparticles (151). By transmitting an inactive or natural adjuvant of antigens and virus particles, vaccines result in a strong immune response.

A novel CD8+ T-cell vaccine based on exosomes was suggested by Lattanzi et al. which depends on the prospects of a dormant mutant kind of the HIV-1 Nef protein (Nef^mut^). The researcher performed a comparison of immunochemical features of Nef^mut^ exosomes with that of lentiviral virus-like particles (VLP) derived from Nef^mut^ and proposed that the strategy of exosomal vaccine derived from HIV-1 Nef^mut^ might accurately subdue key factors concerning production, isolation and safety mostly faced during chimeric VLPs generated vaccine formation. The fact that Nef^mut^-based exosome development does not contain additional retroviral products and lacks anti-cellular effects which turn out new possibilities for the development of untested vaccine approaches (152). Nanjundappa et al. designed a Gp120-Texo vaccine using CD8+ T-cells from ConA-stimulated C57BL/6 mice with exosomes released by a pcDNAGp120-transfected B6 mouse DC line DC2.4 (DC2.4Gp120) in a study. They found that this vaccine provided protective and long-lasting immunity against Gp120-expressing B16 melanoma in both wild-type C57BL/6 and transgenic HLA-A2 mice, suggesting a potential new vaccine for immunocompromised patients with HIV-1 (153). Similarly, Wang et al. developed a Gag-Texo vaccine using ConA-stimulated polyclonal CD8+ T-cells with exosomes released by Gag-expressing adenoviral vector AdVGag-transfected DC. This vaccine stimulated Gag-specific CD8+ CTL responses and antitumor immunity, resulting in protective immunity against Gag-expressing BL6-10 melanoma in both wild-type C57BL/6 and transgenic HLA-A2 mice. This novel immunotherapy vaccine has the potential to benefit HIV-1 patients since HIV Gag is considered a crucial antigen candidate for the development of an HIV-1 vaccine (154).

In another investigation, Wang et al. (155) designed an experimental chronic infection model by injecting C57BL/6 mice with the OVA-expressing adenovirus, AdVova, to evaluate the effectiveness of the novel ovalbumin (OVA)-specific OVA-Texo and HIV-specific Gag-Texo vaccines in promoting therapeutic immunity. The inhibitory molecules programmed cell-death protein-1 (PD-1) and lymphocyte-activation gene-3 (LAG-3) were found to be expressed by chronic AdVova-infected mouse CTLs, and they were also functionally exhausted, exhibiting a marked deficit in T-cell proliferation, IFN-γ production, and cytolytic effects. In mice with persistent infection, naive CD8+ T cells increased inhibitory PD-ligand 1 (PD-L1), B- and T-lymphocyte attenuators, and T-cell anergy-associated markers (Grail and Itch), while downregulating the proliferative response. Surprisingly, the OVA-Texo vaccine converted CTL fatigue and countered T-cell anergy. The latter was connected to (i) an increase in diacetylated histone-H3 (diAcH3), a hallmark for CTL functioning, (ii) a fourfold rise in CTLs occurring independently of host DCs or CD4+ T cells, and (iii) the restoration of CTL IFN-γ production and cytotoxicity. The mTORC1 pathway-related molecules Akt, S6, eIF4E, and T-bet were increased by OVA-Texo-stimulated CTLs *in vivo*. Treatment of the CTLs with the mTORC1 inhibitor rapamycin dramatically inhibited the OVA-Texo-induced rise in CTLs. Interestingly, the observed immunological responses were strongly influenced by CD40L signaling mediated by OVA-Texo. The Gag-Texo vaccine was significant in that it produced Gag-specific therapeutic immunity in patients with chronic illness. Therefore, this research is expected to have a significant impact on the development of novel therapeutic vaccines to combat HIV-1 infection (155).

To induce long-term persistent epigenetic suppression of HIV-1, Scott et al. (156) developed an approach involving the creation of an HIV-1 promoter-targeting Zinc Finger Protein (ZFP-362) fused with the active domains of DNA methyltransferase 3 A. Cells were genetically modified to package exosomes containing RNAs encoding this HIV-1 repressor protein. In humanized NSG mouse models, the researchers observed that these anti-HIV-1 exosomes with repressors effectively reduced virus production, and this suppression was mechanistically driven by HIV-1 DNA methylation. The findings presented in this study pave the way for the development of a platform for the systemic delivery of therapeutic cargo via exosomes, enabling epigenetic suppression of HIV-1 infection (156). Exosomes from various sources have been shown in several studies to potentially contain anti-HIV bioactivity. In this regard, Madison et al. reported the antiviral function of exosomes from healthy human semen, which can prevent the spread of HIV-1 by interfering with viral replication in vaginal epithelial cells. This finding suggests the potential utilization of these exosomes for the development of innovative anti-HIV treatment approaches (157). Naslund et al. (137) demonstrated that exosomes derived from healthy human breast milk exhibit anti-HIV-1 activity by competitively binding to the DC-SIGN receptor, thereby preventing the infection of monocyte-derived dendritic cells (76). In another study, Tumne et al. (157) showed that exosomes produced from CD8+ T cells non-cytotoxically decrease HIV replication by inhibiting HIV-1 LTR promoter transcription (158). To advance these studies, future research should focus on modified exosomes loaded with anti-HIV RNA and target peptides on their surface to achieve highly effective delivery of RNA therapeutics to HIV-infected cells or as a safe adjuvant treatment. Additionally, studies should be conducted to explore new exosome sources with inherent activity against HIV.

In preclinical study trials, it has been seen that exosomes play a crucial function in both pathological and physiological states because they can transmit pathogenic information that suggests disease circumstances. Exosomes might be used as biomarkers for disease and therapeutic efficacy. An investigation of exosomes as biomarkers, in early detection, drug delivery and vaccine development makes them promising agents for clinical use. Researchers believe that exosomes may provide bridges between information gaps in various disease states. The preclinical database shows that exosomes can have applications in the treatment of HIV infection and vaccine-based delivery systems.

## 8 Current challenges in exosome‐based treatment

### 8.1 Heterogeneity of exosomes

When a cell secretes a large number of exosomes with vast biological potential, the process of determining exosome heterogeneity is challenging. Treatments based on exosomes need a better knowledge of the heterogeneity, composition and biogenesis of exosomes (159). In clinical practices, the heterogeneity of exosomes induces extraordinary complications associated with design, delivery system and drug standardization. The heterogeneity of exosomes can be described by the endosomal sorting complex required for transport (ESCRT)-dependent and independent mechanisms, as a major regulator in MVB biogenesis (160). Modifying the properties and sensitivities in detecting methodologies of heterogeneity associated with exosomes is imperative for better knowledge of the characterization of exosomes in both pathophysiological and physiological procedures. This mediates the development of their diagnostic and therapeutic purposes (159). Exosomal subgroups isolated using distinctive extraction procedures are different. This kind of heterogeneity may be effective for the therapeutic purposes of separate exosomes. Therefore, augmenting the extraction procedures is significant to preserve the features of exosomes along with the reduction of the side effects associated with heterogeneity (161).

### 8.2 Choice of cells

**Figure 6:**
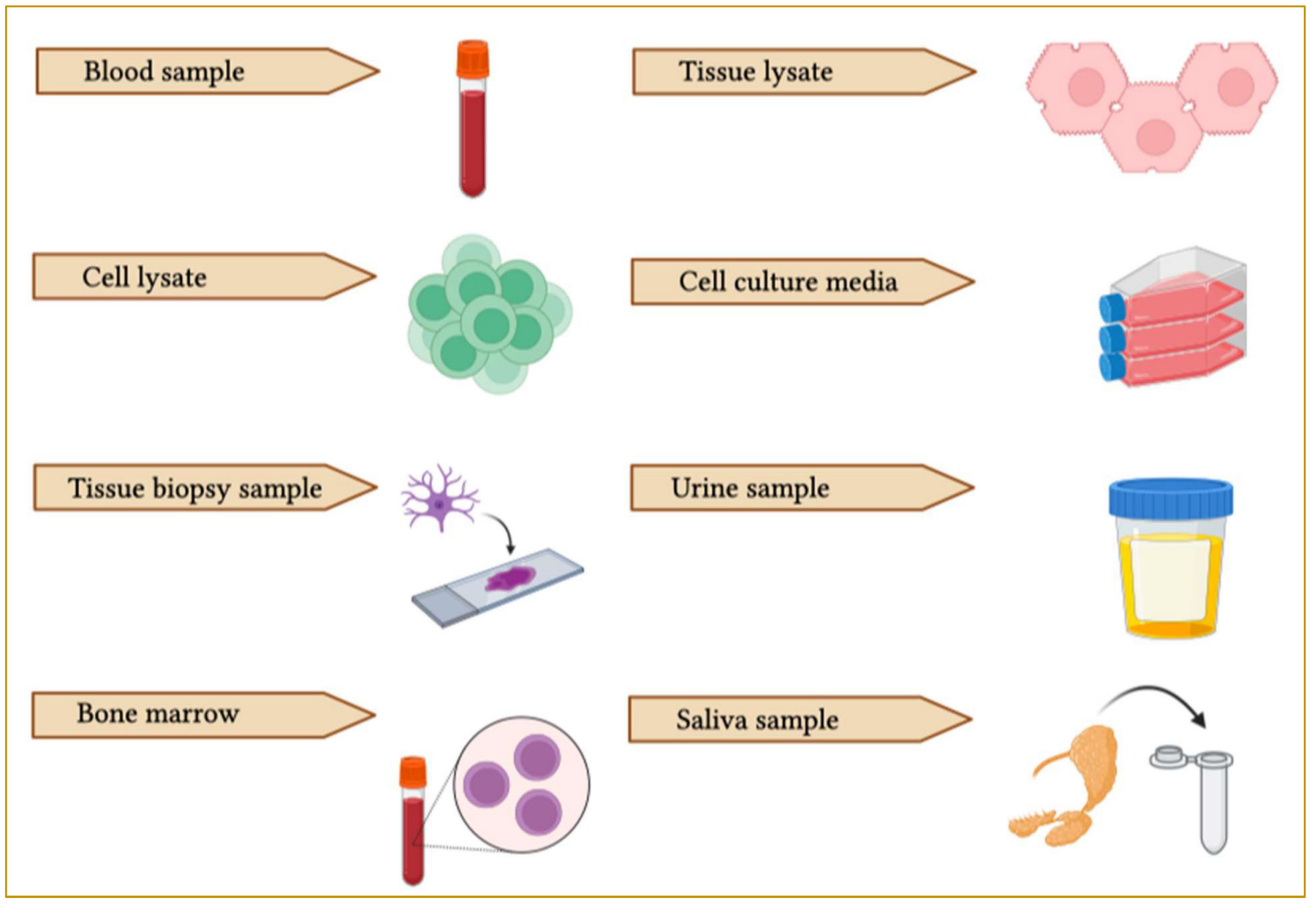
Schematic representation of EV collection sources. EVs can be purified from different biological samples such as blood, tissue lysate, cell lysate, cell culture media, bone marrow, saliva, urine and cerebrospinal fluid (CSF).

Exosomes can be secreted from cells with different functions, but how to select the cells for a particular study is not reported (162). Because of the source and cell-dependent significance of exosomal constituents and surface markers associated with their function, therapeutic practices can be facilitated greatly by the biological potential of exosomes extracted from various cells (163). The *in vivo* biological potential of exosomes is influenced by features associated with parent cells. For instance, evidence suggests the distinctive distribution pattern of exosomes released from melanoma, muscle and bone marrow dendritic cells in the lung, liver and spleen, respectively (164). It was also seen that exosomes derived from neutrophils have the capability of penetrating the blood-brain barrier and may be utilized in drug delivery patterns to gain entry into the target glioma and brain (**Figure 6**) (165). Additionally, the exosomal functional properties depend on the parent cells (166). The usage of exosomes derived from tumors to transmit therapeutic molecules such as anticancer or chemotherapeutic molecules or in vaccine development for immunotherapy can be attractive from various perspectives (167).

Exosomes are secreted from several cells, but the pattern of their secretion and the degree to which they are susceptible varies (168). Exosomes isolated from red blood cells (RBCs) were proposed as a cargo vehicle with different benefits (169). RBCs are cell types without nuclei, therefore a decrease in gene-associated hazards involving horizontal gene transfer is predicted. Additionally, the probability of risks from immunogenic feedback can be reduced by cross-matching blood samples among donors and patients (169). Exosomes isolated from immune cells to be used in drug delivery systems and vaccine development have gained much attention these days. Macrophages and monocyte-derived exosomes showed higher stability by evading phagocytosis which may enhance their efficacy. Also, dendritic cell-derived exosomes have been presented as a way of assisting the rejection of tumor cells by communicating peptide-MHC molecules to other dendritic cells that do not deal with the same antigen (163). Among different kinds of cells producing exosomes, mesenchymal stem cells (MSCs) are also shown to be a powerful exosomal source for clinical approaches because they can be extracted from various tissues and have a huge ex-vivo extension capability (170).

### 8.3 Choosing loading procedures

**Figure 7:**
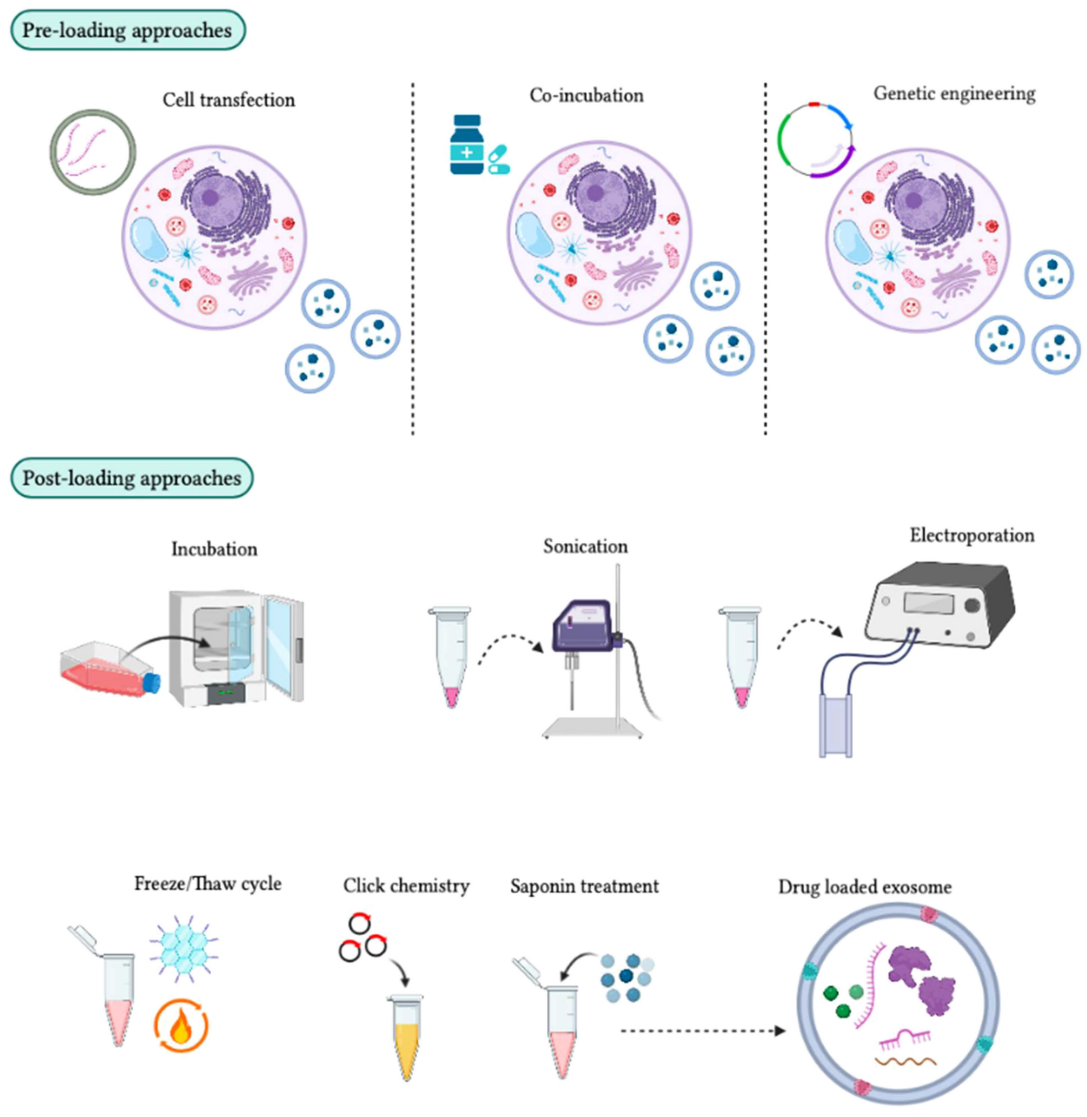
Different approaches for loading therapeutic cargo into exosomes. In the pre-loading technique, therapeutic molecules are integrated into target cells before exosome development. During exosome biogenesis, these molecules are then loaded into the exosomes for therapeutic purposes. The transfection method can be used to package mRNA, siRNA, and miRNA into target cells, although some target cells can passively absorb drug molecules during co-incubation. Genetically engineered exosomes can also be developed by transferring a plasmid into target cells to express drug molecules in the exosomes. In the post-loading technique, therapeutic molecules are packaged directly into exosomes after their isolation using chemical and physical approaches. Chemical methods, such as click chemistry and the saponin method, can be used. Click chemistry involves fusing therapeutic molecules to the outer surface of the exosome membrane, while the saponin method creates pores by interacting with membrane cholesterol. Physical methods, such as incubation, sonication, electroporation, and freeze/thaw cycles, can also be used to enhance exosome membrane permeability for the absorption of therapeutic molecules.

Exosomes designed for specific targets carry various cargoes via genetic engineering. Certain mechanical/chemical procedures can be used to address challenges in the field of biomedicines (171). Several loading plans associated with exosomes extend loading efficacy as well as specifically evaluate the integrity and biological limitations (**Figure 7**) (172). The suitable procedure or new strategy for development purposes should be carefully addressed while remembering its merits and demerits. For instance, several loading strategies and a combination of multiple methods are efficient at enhancing loading capability (172). Although there is a huge progression in the exosomal delivery system, still the mechanism of modification of specific exosomes to increase targeting capacity is unclear and requires investigation (172). The probable hazards associated with exosome modification or changing protein constituents, promiscuous interactions and biological responses must also be addressed (173). In addition, all these loading approaches can be influenced by exosome purity, characteristics and repository state. Hence, future investigations will focus on limiting these factors and examining the therapeutic dose of exosomes in drug delivery systems (174).

### 8.4 Exosome administration route

Exosome’s ability to be internalized by target cells is crucial to their biological efficacy. To fully understand the biological diversity of exosomes for therapeutic applications, several delivery strategies are necessary to consider, such as their pharmacological potential, physiological distribution, and rapid clearance (47). Moreover, the storage of administered exosomes in the spleen, liver, and lungs may be attributed to high vascular permeability resulting from inflammation and injury. PEGylation can overcome this disadvantage by preventing quick exosomal clearance from circulation (48). Local and direct administration of cargo exosomes with targeted or therapeutic agents is an appropriate injection strategy for delivering therapeutics to targeted sites. The intraperitoneal route provides a high dosage of loaded exosomes, but because of the increased area of the peritoneal cavity, administered exosomes quickly dilute and distribute in more distant localities (48). Even though oral introduction is convenient and easy, enzymatic activity, gastrointestinal barrier, gastrointestinal microflora and severe acid-base changes are issues related to the delivery of exosomes to targeted sites. Intranasal administration is a highly efficient procedure, that addresses the problems associated with drug delivery systems in the whole blood-brain barrier. This strategy prevents the hepatic and intestinal metabolism of exosomes, thereby conserving the vesicles of exosomes in brain tissues (48). Non-invasive introduction through inhaling is one of the most efficient strategies for therapeutic molecules for several lung disabilities. The efficiency of this potential methodology is closely associated with the feature and quantity of drug absorption by recipient cells, mucociliary clearance, breathing patterns and respiratory tract configuration (49).

## 9 Limitations of exosome research in the clinical setting

With considerable development in this area over the last few decades, exosomes have brought intense probabilities for diagnosis and therapies for various fatal diseases. Even though treatments are under consideration, the exact mechanism of biogenesis is still unclear. Purification technologies for exosomes are laborious and inflexible in clinical approaches. Researchers evaluated several commercial kits available for exosome extraction but they still need to conduct further investigations into this prospect. Researchers cannot authenticate potential outcomes unless we have unified characterization and purification procedures in place. However, due to complications and deviations in strategies, the reproducibility of the exosomes is vastly deviated, which shows complexity in the elucidation of results. Hence, we require standard operating procedures (SOPs) for isolating exosomes, accumulation, analysis and characterization. With the intense understanding of functions and biogenesis, exosomes will significantly open up new horizons in HIV-1 treatments and recent studies have already examined exosomes as an HIV-1 biomarker and natural gene/drug delivery system. To conclude, EV field urgently require effective separation strategies in this research area.

## 10 Conclusion

During HIV-1 infection, intense stimulation of the immune system results in the severe loss of immune cells, leading to a sustained and persistent inflammatory environment that ultimately causes chronic inflammatory activation. Immunotherapy to control disease progression is challenging. It has been demonstrated that HIV can influence how miRNA networks are regulated and modulated, affecting how infected cells respond to the virus. HIV progression in positive individuals is linked to miRNA dysregulation. A greater understanding of the underlying cellular and molecular mechanisms involved in HIV pathogenesis will be possible through continued research, facilitated by the discovery of miRNA sequences in HIV-positive individuals. Furthermore, miRNAs might be used as diagnostic biomarkers for tracking HIV-positive individuals. However, the role of exosomes in HIV-1 pathogenesis is an advancing field with extensive therapeutic approaches aimed at HIV-1 remission. In the current framework, engineered exosomes could be used as delivery systems for therapeutic molecules targeting cells and tissues. Different studies have shown that exosomes from breast milk and semen have anti-HIV activity. Therefore, the assessment of exosomes isolated from different biological sources in infected patients could be used to monitor HIV-1 progression. Further *in vivo* experimental analysis is urgently required to regulate the role of exosomes in HIV-1 infection and their potential use in drug delivery systems in clinical settings. New insights into the function of these immunomodulatory nanovesicles in AIDS pathogenesis will emerge from the identification of the biomolecules carried by exosomes and the clarification of their immune regulatory impact in HIV-positive patients. By gaining a better understanding of the potential role of exosomes in HIV-1 infection, we may be able to develop novel strategies for combating this deadly virus.

## Author contributions

AH: Conceptualization; Data curation; Formal analysis; Investigation; Writing-original draft; Writing-review & editing. YL: Data curation; Formal analysis; Investigation; Writing-review & editing. NZ: Investigation; Project administration; Supervision. All authors contributed to the article and approved the submitted version.
